# 20 Years nitrogen dynamics study by using APSIM nitrogen model simulation for sustainable management in Jilin China

**DOI:** 10.1038/s41598-021-96386-5

**Published:** 2021-09-01

**Authors:** Nazia Tahir, Jumei Li, Yibing Ma, Aman Ullah, Ping Zhu, Chang Peng, Babar Hussain, Subhan Danish

**Affiliations:** 1 Institute of Agricultural Resources and Regional Planning, Chinese Academy of Agricultural Sciences, 12 Southern Street of Zhongguancun, Beijing, 100081 China; 2 Department of Agronomy, Gardan Campus, Abdul Wali Khan University, Mardan, Khyber Pakhtunkhwa Pakistan; 3 Macau Environmental Research Institute, Macau University of Science and Technology, TaipaMacau, 999078 China; 4 Jilin Acadmey of Agricultural Science, Jilin City, China; 5 Department of Soil Science, Faculty of Agricultural Sciences and Technology, Bahauddin Zakariya University, Multan, 60800 Punjab Pakistan

**Keywords:** Climate sciences, Environmental sciences, Environmental impact

## Abstract

The tremendous increase in industrial development and urbanization has become a severe threat to the Chinese climate and food security. The Agricultural Production System Simulator model was used to simulate soil nitrogen in black soil in Yangling Jilin Province for 20 years. The observed values are consistent with the simulated values. The predicted values of total soil NO_3_^−^–N and NH_4_^+^–N nitrogen are 10 kg ha^−1^ and 5 kg ha^−1^ higher than the observed values. The total soil NO_3_^−^–N loss has the same trend as the rainfall, and it increases with the number of rainfall days over the years. The average 20 years losses of NO_3_^−^–N and NH_4_^+^–N observed were 1375.91 kg ha^−1^, and 9.24 kg ha^−1^, while in the simulation increase was 1387.01 kg ha^−1^ and 9.28 kg ha^−1^, respectively. The difference between the observed and simulated values of NO_3_^−^–N and NH_4_^+^–N of mean loss was 11.15 kg ha^−1^ and 0.04 kg ha^−1^ respectively. Moreover, our findings highlight the opportunity further to improve management policies (especially for nitrogen) to maintain crop yield.

## Introduction

Global agricultural intensification significantly increased food production over the last decade, driven by increased fertilizer use and irrigation^1^. By 2050 the world will need 70–100% more food^2,3^, China must feed 20% of the world’s population with 7% of the world’s arable land and 6% of its water resources. A recent study on global climate change has shown significant changes by warming^4^. The intergovernmental panel on climate change (IPCC) fifth assessment reported that the earth surface temperature had been continuously increasing in the past three decades. Around 1880 and 2012, the global average temperature increased by 0.85 degrees Celsius, and the temperature rise from 1951 to 2012 was nearly double that of 1880–1950^5^. The regional and global weather conditions are expected to become more change than present, with the increasing flood, hailstorm, drought, and other climatic changes^6^. By bringing in greater crop yield variability, local food supplies, and higher landslide and erosion damage risks, they can adversely affect food supply stability and food safety. In addition, the rates and levels of the predicted warming may increasing the historical experience in some regions^6,7^. China has encountered one of the most significant challenges in the twentieth century in trying to maintain increased annual cereal production to about 600 Mt by 2030 and to assure food security with diminishing cropland and limited resources while improving soil fertility and protecting the environment. Chinese farmers have been switched from a traditional farming system to advance which was accumulated with efficient utilization of different crop rotation, intercropping, and all possible nutrients (e.g., nitrogen (N), phosphorus (P), potassium (K), and Sulphur (S) resources to the usage of synthetic fertilization^8^. So, there is an urgent need for long-term field experiments to understand productivity, soil nutrients, crop management, weather processes & interactions, and to maintain sustainable agricultural management in the future. Some trends of soil processes cannot be consistently determined during short-term studies and may be visualized over a period of time^9^. Disproportionate and uninterrupted addition of ammonium or urea-based N fertilizers in crop production caused soil acidification. It also changes soil chemical properties^10^, that can minimize biodiversity, hinder nutrient cycling and release potentially lethal metals into water and plants, leading to environmental pollution, soil degradation and reduced crop yield^8,9,11–13^. Soil type and topography are relatively constant within time, while land use and vegetation are influenced by anthropogenic activities and climate^14^. Therefore, China had big challenge with food security^15^. Agricultural production plays a significant role in global climate change^16^. China’s food production is highly dependent on irrigation, but water scarcity has challenged irrigated agriculture^17^. Agricultural production has a significant impact on global climate change^16^. Approximately 65% of global nitrous oxide (N_2_O) emissions are from agricultural soils. this primarily comes from nitrogen (N) fertilizer, which is misused at the national level and the optimization of N management, therefore, a target for sustainable agriculture^18^. Furthermore, large inputs of mineral N fertilizer above crop demands can result in low N use efficiency and lead to several negative impacts on the environment, such as surface water eutrophication, groundwater nitrate pollution, soil acidification, and emitting greenhouse gases. Such environmental problems are getting worse because mineral N fertilizer will increase predictably in the future^19^.

APSIM (Agricultural Production Systems Simulator) is a software system that provides a versatile framework for simulating climate and soil management effects on crop growth in agricultural systems and soil resource changes. APSIM’s predictive accomplishment for soil water and nitrate nitrogen simulation in contrasting soils (clay loamy) and environments^20^. APSIM allows modular configuration of crop models, pastures, soil water, nutrients, and erosion to simulate the different production systems^21^. Many variables, including weather fluctuations, will restrict producers' revenues and reduce yield by moderate inputs^22^. A key feature of APSIM, which differentiates it from other models, is the soil’s central position rather than the crops. In response to management and weather, alteration in the status of soil state variables are simulated crops constantly come and go, find the soil in a given state and leave it in a changed state. Another aspect is its integrated structure: high order mechanisms (i.e., soil water balance, crop production and soil N dynamics) as distinct modules. Several process-based models are in use or are being developed, for example, APSIM^23^, DAISY^24^, DNDC^24^, and WNMM^25^. These models present the ability to examine the role of particular process towards system outputs. They help to understand better that how environmental conditions shared with management strategies interrelate to control N cycling and losses. However, the conceptualization of N transformations in APSIM and DNDC models are different. DNDC is a microbial growth model. APSIM defines denitrification and nitrification processes using equations of empirical reaction conveyed using Michaelis Menten type equation^26^. APSIM model correlated well with measurements (*r* = 0.97), while NZ-DNDC performed well on the Otago soils (*r* = 0.83 and 0.92 for Wingatui and Otokia, respectively^24^. Much of the code that comprises the APSIM SOILWAT and soil nitrogen has developed from prior experiences with, firstly, models of the CERES family, notably CERES-Maize^27^ and, secondly, PERFECT^28^ was primarily established to create the effects of erosion on productivity of vertisols in the Australian subtropics but did not address N. PERFECT involved schedules for simulating impacts of surface residues on soil runoff and evaporation. Surface residues decay was modelled as a simple time function. The CERES models treat and residues from previous crops as integrated into the soil and therefore cannot involve any effect on soil water balance from surface residues. CERES deals with nitrogen, and the simulation of fresh residue decomposition takes into account the residue ratio C: N and the moisture and temperature environmental factors. These models' code had been re-engineered into discrete modules. APSIM was a broadly used model of the agro-ecosystems. It stimulates plant growth on a daily basis, based on incoming solar radiation and depends on both water content and Nitrogen supply. In APSIM the standard model for soil, water uses a tipping bucket approach^29^. It simulates nitrate movement linked to the vertical water flow, and eventually drainage and leaching. Evapotranspiration potential is calculated using Priestley Taylor^27^ and the runoff is calculated using an approach to the USDA soil curve number.

This study aimed to study the dynamics of nitrogen fertilization and its effect on nitrification and denitrification. The vertical movement of nitrate in soil was investigated. To explore the novelty of the simulation, to observe and predict future nitrogen loss under the same conditions of nitrate content change, to reduce the 20-year environmental consequences and its impact on nitrogen loss, the nitrogen model was calibrated and validated. New to this study is examining nitrogen dynamics and its response to the environment and the use of the APSIM nitrogen model**.**

## Materials and methods

### Site description and experimental data

Data for the study was measured from the long-term soil monitoring research station in Gongzhuling-Jilin, China. The province is located between (43° 30′ 23′ N, 124° 48′ 33′ E) latitude and longitude while the altitude of (220 m) the study area in China. Jilin's mean annual temperature and precipitation were found as 4–5 °C & 500 mm with cool temperate, highly continental, and 13.5 °C & 1000 mm respectively. The crop was maize and which holds a relatively large area and main crop. The soil type is clay loam, and pH was 7.6. The organic carbon C (g kg^−1^), Total N (g kg^−1^), Total P (mg kg^−1^), Available N (kg ha^−1^), Available P (mg kg^−1^), Available K (mg kg^−1^) were 20, 1.34, 0.546, 15.3, 10, 119. Soil pH was measured in a 1:5 soil: ratio, the organic carbon g kg^−1^ was measured by the walkley wet combustion method^30^, total N measured by the Kjeldahl method^31^, available P with 0.5 M NaHCO_3_^32^, and exchangeable K with N ammonium acetate^33^. The long-term experimental sites in Gongzhuling, Jilin, China was measured from 1990 to 2010. To check the -long-term status on the soil nitrogen dynamic and s improve this soil better for estimating the pollution level and crop productivity. A composite soil was selected for the soil sampling, and the soil was sampled in October every year, up to the depth of 20 cm, and for APSIM modeling, simulation depth was kept at up to 180 cm. The APSIM soil parameters include soil property and hydrodynamic values; these values were calculated by the formula. These values were used by APSIM nitrogen model to simulates soil properties and nutrient cycling by each soil layer.

### Weather module in APSIM

Daily weather data were collected from a weather station 50 m away from the experimental site, including daily maximum temperature (Tmax), minimum temperature (Tmin), sunshine period, and precipitation between 1990 and 2010. The sunlight period was transformed into solar radiation using the Ångstrom formula^34^.Historical weather data were downloaded from China’s meteorological sharing service network (http:/cdc.cma.gov.cn/). The essential module for the simulation of the APSIM model is the meteorological module (APSIM met generator). Temperature (Tav), the maximum amplitude of the monthly average temperature (Amp), time (year and day); Tav and Amp for each location were calculated by the software included with APSIM 7.1^35^. The weather file was created by following the below link in APSIM https://www.apsim.info/support/apsim-training-manuals/creating-an-apsim-met-file-using-excel/.

It runs daily weather data as an input file, i.e., daily maximum and minimum temperature, rainfall, and solar radiation. The Tav (annual average ambient temperature) is about 5.82 °C, and Amp (annual amplitude in mean monthly temperature) is 39.5 °C. The line graph with variation showed the minimum recorded amount of yearly precipitation. The total precipitation from 1990 to 2010 is 17,560.1 mm. The total precipitation was 587.3 mm in 1990, 251.22 mm in 1995, 283.8 mm in 2000, 334.7 mm in 2005, and 389.3 mm in 2010. The regression analysis over 20 years with the precipitation was (r^2^ = 0.9977) maximum temperature (r^2^ = 0.0001) and minimum temperature was (r^2^ = 5E−05). The Precipitation trend analysis showed that the (r^2^ = 0.997) over 20 years and significantly changed was observed in Table S5.

### APSIM model overview

APSIM includes a series of modules that simulate biological and physical processes in farming systems, including crop growth, soil water, and carbon and nitrogen dynamics due to climate variation and interaction^20^. This analysis performed in APSIM version 7.5. Maize crop production is measured as a function of photoperiod-modified thermal time (Table 1). The Agricultural Production Systems Simulator (APSIM) is a modular modeling framework that has been developed by the Agricultural Production Systems research unit in Australia^36^. Four modules, a soil–water module (SOILWAT2), the soil nitrogen module (SOILN2) and the fertilizer module (FERTILIZ), and a specific crop module (APSIM-maize), were linked within APSIM to simulate the cases described in this paper (Table 2). APSWIM is based on the numerical solution of the equation of Richards coupled with the formula of convection dispersion to the movement of the model solute. The APSIM model’s application is based on the ‘standalone’ SWIMv2.1 (soil water infiltration and movement)^37^. Soil water properties parameterization for APSWIM includes specification of the relationships of moisture characteristics and hydraulic conductivity in each soil layer. Runoff is handled by considering the roughness of the soil.

**Table 1:** List of APSIM Modules used in the study and simulate in the nitrogen model.

S. no	Modules	Abbreviation of Model	Modle name	References
1	Soil water	SoilWat	CERES	^35^
			PERFECT	^28^
2		APSWIM^a^		^42^
3	Soil nitrogen	SoilN	CERES	^43^
4	Maize	AUSIM maize		^44^

Intellectual property remains that of the original developer. APSWIM (APSIM-Soil water infiltration and movement) it measures soil water behavior in APSIM.

^a^ Four modules, a soil–water module (SOILWAT2), the soil nitrogen module (SOILN2) and the fertilizer module (FERTILIZ), and a specific crop module (APSIM-maize).

**Table 2:** Crop’s LL = lower limit (water content at -15 bar pressure potential), KL = rate of water extraction XF parameters of the APSIM model at Jilin site.

Soil layers (cm)	1	2	3	4	5	6	7
Maize LL (mm/mm)	0.11	0.14	0.16	0.17	0.19	0.2	0.2
Maize KL (/day	0.08	0.08	0.08	0.08	0.06	0.04	0.03
Maize XF (0–1)	1	1	1	1	1	1	1

There are seven layers in from 0 to 180 cm.

**Figure 1:**
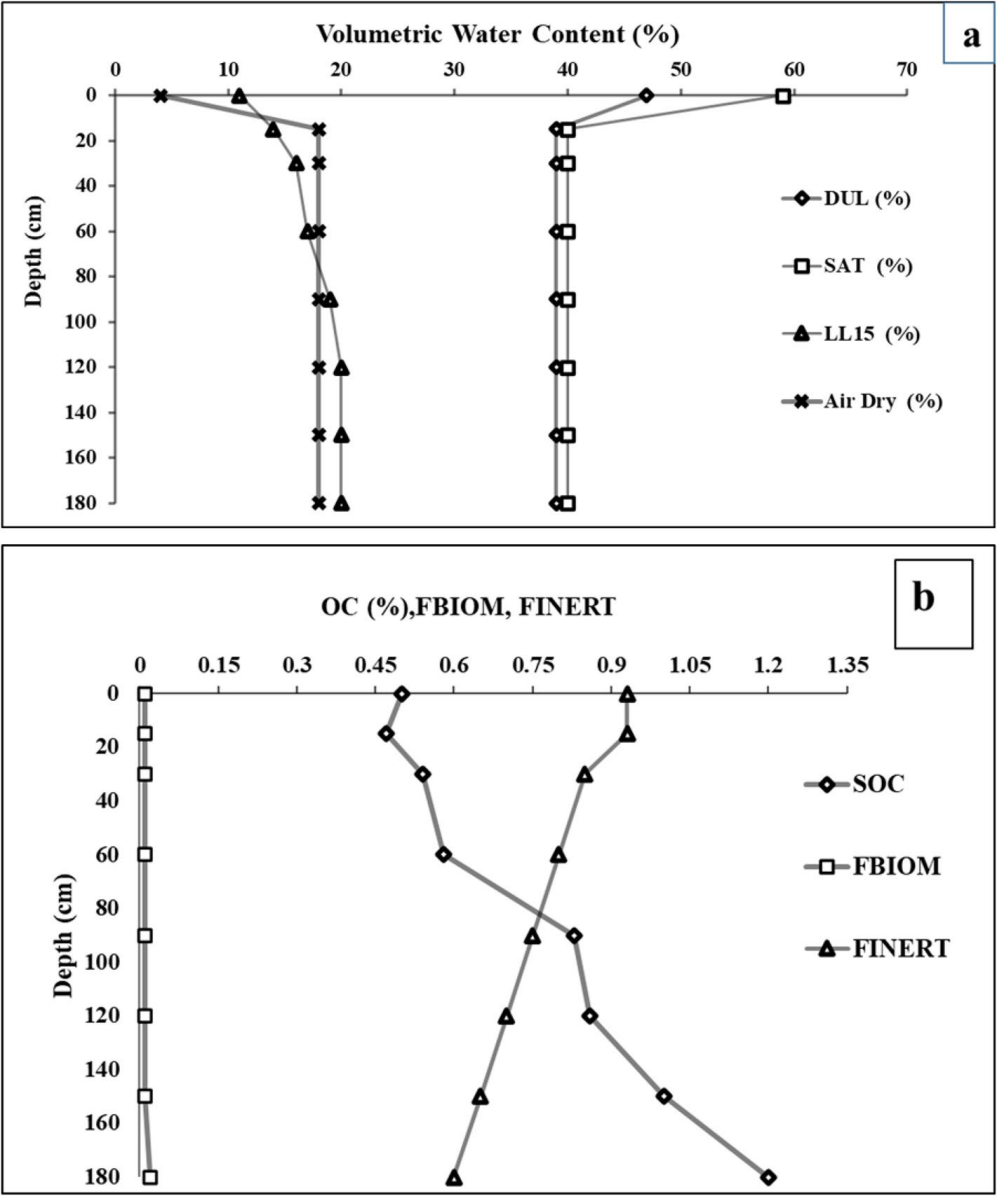
(**a**) Volumetric water content and whereas (SAT) is the saturated water content, (DUL) is the field water holding capacity and lower water absorption limit (LL15) (**b**) Soil SOC (soil organic carbon), fraction of biomass_C, inert_C,and volumetric water content in soil profile in Jilin. The data was from initial soil sample collected from Jilin.

**Figure 2:**
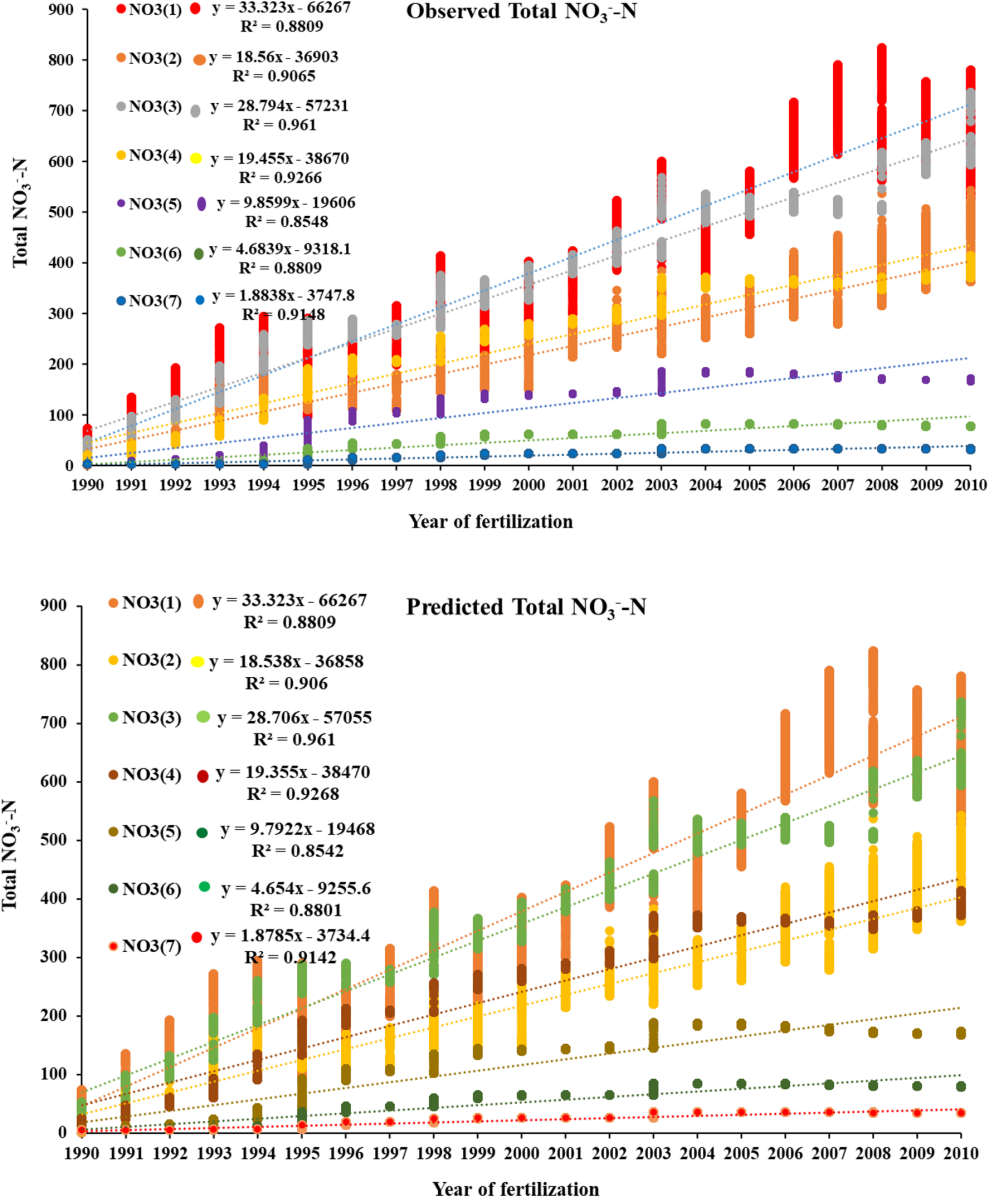
The APSIM observed and simulated NO_3_^−^–N of 7 layers of the soil under the same weather condition with different ammonia and nitrate levels at Gongzhuling, Jilin. The R^2^ values showed significant change among the years over 20 years in predicted and observed data.

The ability to hold surface water might be change over time, e.g., increasing due to agricultural practice or decreasing due to raindrop effects. The surface roughness of soil also effects rainfall effect. This also varies in response to tillage performance. The soil–water module (SOILWAT2) is a model of water balance in cascades^23,29^. Water movement is defined as using separate saturated or unsaturated flow algorithms^23^. In this module, the redistribution of solutes, such as nitrate- and urea-N, is done. Evaporation is a two-stage process based on potential evaporation (Priestly-Taylor) (energy-limited and water-limited). The APSIM model is distinctive to soil organic matter. The CERE model divided the soil organic matter into two subgroups: fresh organic matter (FOM) and humus (HUM), which boosts the microbial pool. HUM, is not susceptible to decomposition; this is specified as Finert (Figs. 1a, 2b).

Although soil microbial biomass only represents a small part of organic matter, it has a significant effect on the nutrient cycle and soil nitrogen^38,39^. The APSIM soil pH module provided a representation of soil acidification and how pH changes are disseminated through the profile due to cation and anion absorption imbalances, nitrate leaching, and changes in soil organic matter content^40^ and soil pH refers to the proton balance^41^. APSIM's evolution was primarily as a modeling framework for climate response and management simulation of cropping systems. Soil nitrogen is the module that simulates nitrogen mineralization and thus the N supply available to a soil crop and residues/roots from previous crops^29^. The model observed value for NO_3_^−^–N was 15 kg ha^−1^, and NH^+^–N was 0 kg ha^−1^, while in predicted NO_3_^−^–N was 25 kg ha^−1^ and NH_4_^+^–N was 5 kg ha^−1^. Fertilizer amount N, P, and K at a soil monitoring station in Gongzhuling, Jilin from 1990 to 2010 for NPK treatment N kg ha^−2^ was 165 kg ha^−2^, P_2_O_5_ 82.5 kg ha^−2^, and K_2_O 82.5 kg ha^−2^. The Control has no fertilizer.

### APSIM parameterization

The soil module parameterization is critical to correctly simulate the nitrogen and water balance of the soil–crop system. The soil textures, drained upper limits (DUL) and lower limits (LL15) obtained from laboratory measurements were used to set these parameters. The initial water content was selected to the soil water storage capacity. The crop was maize, and the soil organic carbon and nitrogen value is written in the site description in the material method. The initial surface residue was zero, and the organic matter pool name was maize.

### Vertical movement in the soil profile of nitrate

To check the distribution of nitrate through the soil profile, throughout the depth after 31 days of fertilization, and again after five months. To visualize this, we created a depth plot. Two values needed for depth plot, the “dlayer” variable. It holds the depth of each variable. The layered variables were always kept in arrays form. We included NO_3_^−^–N and NH_4_^+^–N as a variable (layered) and not as total.

### Soil parameters in APSIM

There are two soil parameters such as hydrodynamic parameters and soil property. The different models in APSIM simulate nutrient cycling and soil properties by soil layers. These various soils can also give of different results and all the changes that happened in the layers. This obliged the study of the vertical soil distribution of nitrogen and water dynamic changes in the complete soil profile and root zone. The average saturated water content of the 0–180 cm soil depth is 59.1%, the field water holding capacity is 47%, and the soil bulk density is 1.07 g cm^−3^. The soil organic carbon, soil microbial biomass, and passivated organic matter was shown in Fig. 1a, and the soil hydrodynamic parameters are shown in Fig. 1b. The soil properties were modified based on the measured values at other locations. The hydrodynamic parameters are calculated in conjunction with the parameters of the soil properties. Equations 1 are as follows:

1 $${\text{SAT}} = 1 - {\text{BD}}/2.65 - 0.05$$

here SAT is the saturated water content, DUL is the field water holding capacity, BD is the soil bulk density, and 2.65 is the soil-specific gravity. The soil pH was 7.6; bulk density was 1.07 and change to 1.35 up to the depth of 180 cm. The total depth was divided into seven layers. SAT and DUL reduced with the depth from (59 to 49) % and (47 to 39) %. Depth layer is the soil layers divided into seven layers from top to bottom as shown in Fig. 1a.

### Crop parameters in APSIM

The crop in the study site was maize. The maize module also involves two crops, soil parameters, namely the crop’s lower water absorption limit (LL) and the crop water absorption coefficient (KL) at various root depths. LL characterizes the residual value of crops after different soil water depths after the water supply was discontinued during the vigorous crop growth period. KL characterizes the day-to-day absorption of effective moisture by crop layer. Since there was no measured value of LL, LL15 was used instead of LL, and only KL is adjusted. Since there was no moisture calculation in this experiment, KL’s estimate was based on the APSIM estimation process and value in Australia (Table 3).

**Table 3:** 20-years average soil NO_3_^−^–N and NH_4_^+^–N, essential soil water and urea and denitrification losses in the soil layer from 1 to 7 in observed and predicted values.

S. no	Observed	Predicted	Change in observed and predicted
Essential soil water	286.73	286.73	0.00
Total NO_3_^−^–N (kg ha^−1^)	1375.86	1387.01	11.16
Total NH_4_^+^–N (kg ha^−1^)	9.24	9.28	0.04
NO_3_^−^–N (1) (kg ha^−1^)	378.65	379.04	0.39
NO_3_^−^–N (2) (kg ha^−1^)	217.42	217.75	0.32
NO_3_^−^–N (3) (kg ha^−1^)	356.63	357.70	1.07
NO_3_^−^–N (4) (kg ha^−1^)	239.69	241.37	1.67
NO_3_^−^–N (5) (kg ha^−1^)	113.89	116.12	2.23
NO_3_^−^–N (6) (kg ha^−1^)	49.74	52.36	2.62
NO_3_^−^–N (7) (kg ha^−1^)	19.83	22.68	2.84
NH_4_^+^–N (1) (kg ha^−1^)	8.49	8.49	0.00
NH_4_^+^–N (2) (kg ha^−1^)	0.30	0.30	0.00
NH_4_^+^–N (3) (kg ha^−1^)	0.29	0.30	0.01
NH_4_^+^–N (4) (kg ha^−1^)	0.16	0.16	0.01
NH_4_^+^–N (5) (kg ha^−1^)	0.00	0.01	0.01
NH_4_^+^–N (6) (kg ha^−1^)	0.00	0.01	0.01
NH_4_^+^–N (7) (kg ha^−1^)	0.00	0.01	0.01
Denitrification (kg ha^−1^)	0.06	0.06	0.00
Urea (kg ha^−1^)	3.58	3.58	0.00

### Statistical analysis

#### Model calibration and validation

To test the accuracy of modeled, soil nitrogen data was compared with observed and simulated data and correlate with various measures such as^45^. These included the (R^2^) and (RMSE) coefficient of determination, root mean square error. To determine model performance, we calculated the following four indicators: coefficient of determination (R^2^), Root Mean Square Error (RMSE), and index of agreement (D-index). Willmott concordance index (d) describes the deviation between observed and predicted values. If d = 1, it means that the model performance is perfect while d = 0, it indicates a model for which the mean square error is just matching the variability in the observed.

2 $${\text{RMSE}} = \sqrt {\mathop \sum \limits_{{{\text{i}} = 1}}^{{\text{n}}} \left( {{\text{O}}_{{\text{i}}} - {\text{pi}}} \right)^{2} /{\text{n}}}$$

3 $${\text{d}} = 1 - \frac{{\mathop \sum \nolimits_{{{\text{i}} = 1}}^{{\text{n}}} \left( {{\text{Oi}} - {\text{Pi}}} \right)^{2} }}{{\mathop \sum \nolimits_{{{\text{i}} = 0}}^{{\text{n}}} {\text{IPi}} - {\text{IOi}} + {\text{IOi}} - {\text{OI}}))^{2} }}$$

In our study, the crop was maize, the average sowing date and maturity date was observed from the field of long-term of Gongzhuling (43° 30′ N, 124° 48′ E), it was validated with^46^ dates. Calibration is a significant step for the adjustment of model parameters under location agro-climatic conditions. The model calibration of the phonological parameters of maize, sowing dates, and harvesting dates were observed values of the site. The model was run when soil and weather data of the site was input and the year of the simulation was from 1990 to 2010. The model was parameterized through adjustment of soil and crop and weather file factors for maximum matching of observed and simulated data. The field result was used for the model calibration from the 1990–2010 field experiment with model outputs. In this step, for the parameterization of the models, we used the derived parameter. The process of calibration followed phases: soil data, climate data, fertilizer data, and crop data. We then used the supplementary data set to independently validate the models. For calibration and validation, we analysed the goodness-of-fit among models observed and simulated the values of nitrogen and total nitrate and total ammonia as well as denitrification. In addition, the conventional R^2^ regression is for comparison calculation of the mathematical (least-squares decision coefficient), which is fundamental, which is vital when testing simulation model output.

### Regression

We used SPSS to check the regression model for estimating the climatic change in precipitation, temperature. We also used trend analysis to check the change in 20 years.

### Plant material collection and use permission

No permission is required for plant material as it was purchased from certified dealer of local area.

### Ethics approval and consent to participate

We all declare that manuscripts reporting studies do not involve any human participants, human data, or human tissue. So, it is not applicable.

### Complies with international, national and/or institutional guidelines

Experimental research and field studies on plants (either cultivated or wild), comply with relevant institutional, national, and international guidelines and legislation.

## Results

### Model performance

**Figure 3:**
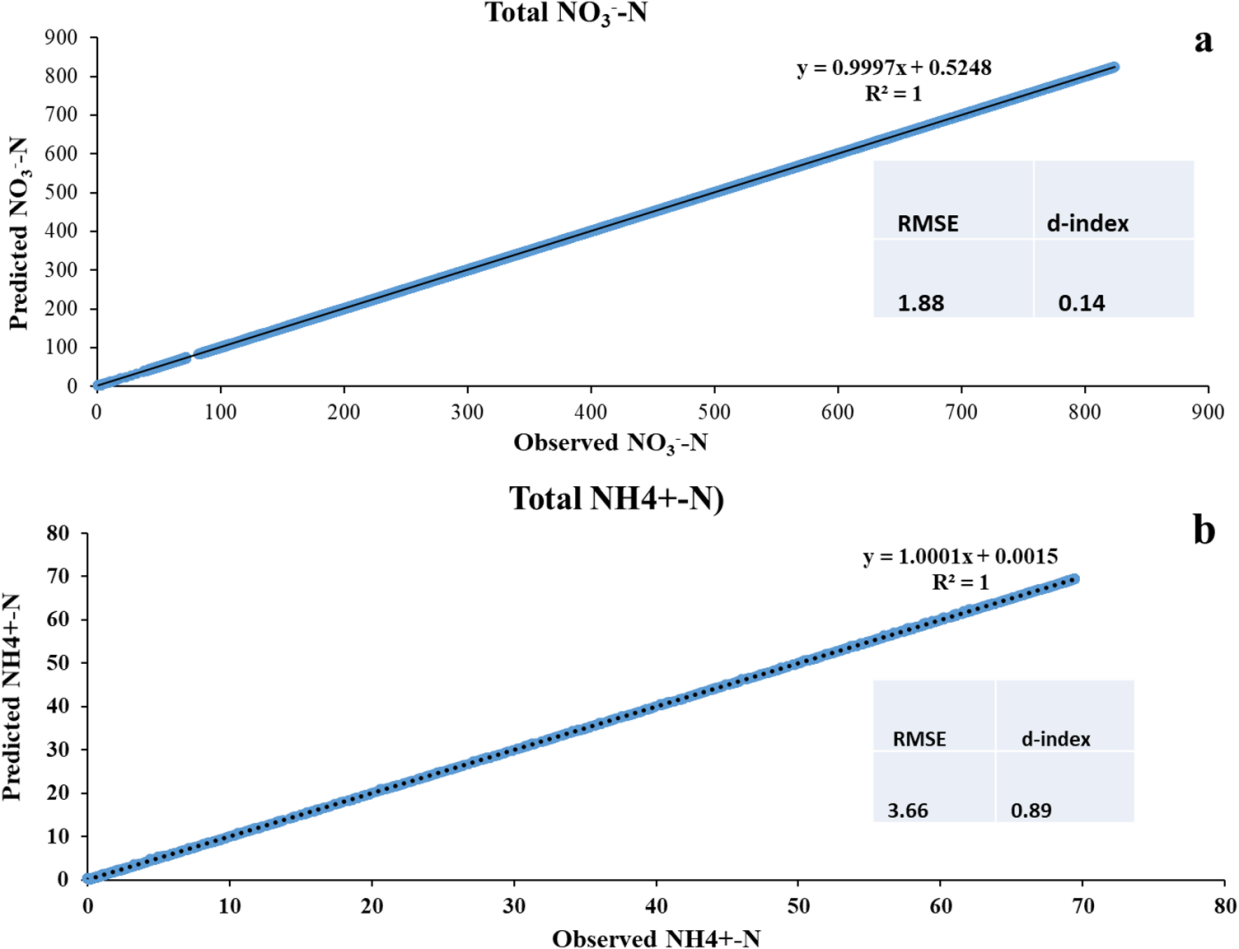
(**a**) APSIM total NO_3_^−^–N observed and simulated with R^2^ value and RMSE. where R^2^ represents the coefficient of determination and RMSE (root mean square error). (**b**) APSIM observed and simulated total NH_4_^+^–N with two different scenarios under the same weather condition and fertilizer inputs. The observed and simulated changes over 20 years of fertilization observed and predicted total NH_4_^+^–N calibration with R^2^ and RMSE (root mean square analysis).

**Figure 4:**
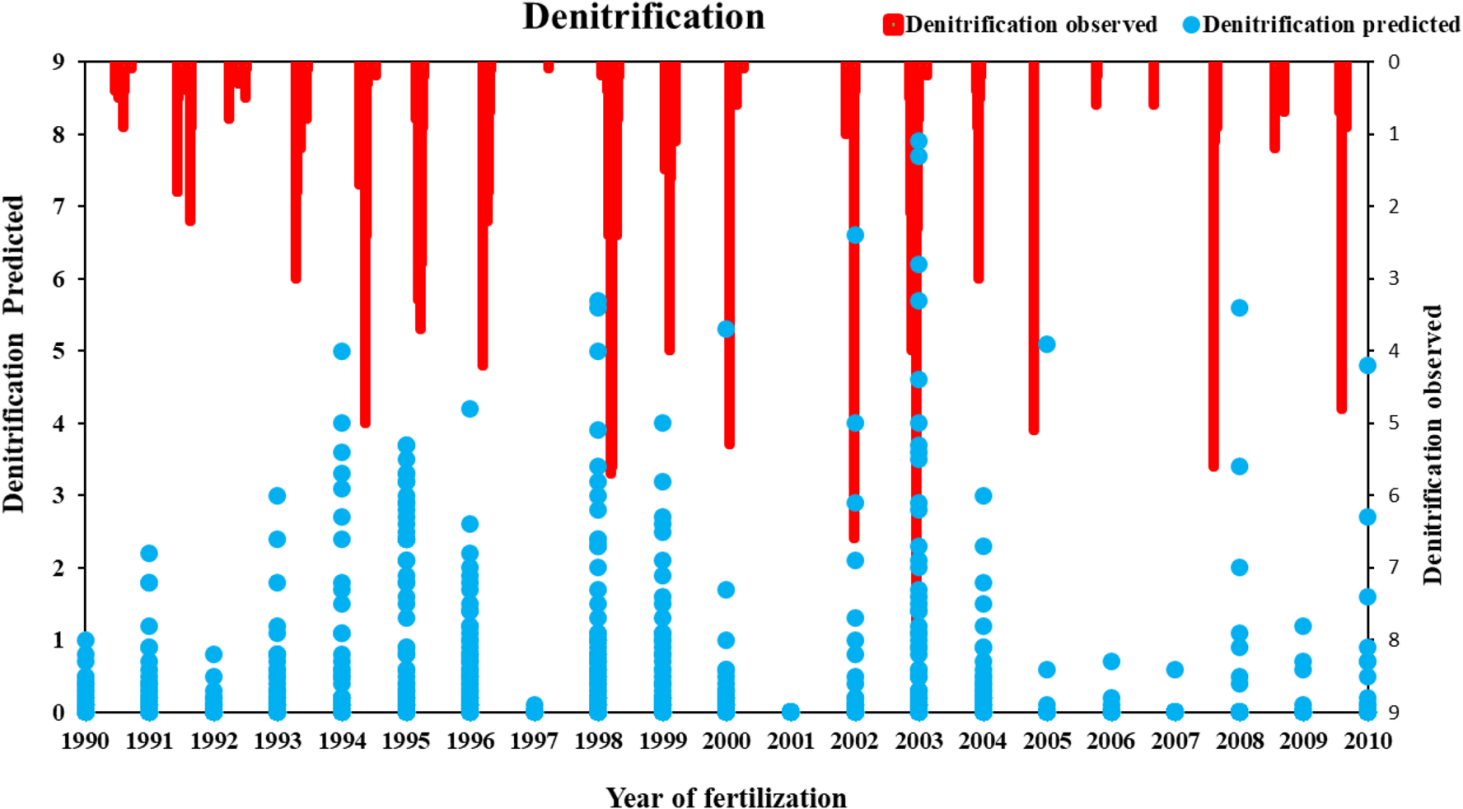
Denitrification rate over 20 years of fertilization of predicted and observed values.

The performance of APSIM model calibration in terms of maize simulation, N dynamic, soil water, and losses of NO_3_^−^–N and NH_4_^+^–N from the soil zones shown in Figs. 1a, 2, 3 and 4. APSIM was able to capture the maize crop by adjusting the soil and climatic phenomena. Comparison of soil N dynamics, NO_3_^−^–N, NH_4_^+^–N predictions and observations in simulated maize season. APSIM can capture maize crops by adjusting soil and climate phenomena. The model also correctly simulates the dynamics of NO_3_^−^–N and NH_4_^+^–N, as shown in Fig. 4. Calibration model parameters were based on nitrogen processing using observations of NO_3_^−^–N and NH_4_^+^–N and RMSE (RMS error) is 1.889, (d-index) 0.14 is 0.14, ammonium-NO_3_^−^N, R^2^ is 1, RMSE is 3.67, d index is 0.895 (Figs. 2, 3, 4). Observations of denitrification in both observed and simulated conditions show that losses were low high in predicted as compare to observed. The losses were not significantly different because the percent change was low (Fig. 4).

### Total NO_3_^−^–N and NH_4_^+^–N changes over 20 years of fertilization

During 20 years of fertilization, soil changes of NO_3_^−^–N were observed and simulated. A comparison between the observed and simulated results showed that simulated NO_3_^−^–N had higher variations in fertilization and loss. The average loss of NO_3_^−^–N depends on the application of nitrogen input to the soil system. The simulation was done on seven soil layers, and each layer showed significant changes with the year of fertilization. Regression analysis showed that the significant change over 20 years in observed and simulated. The average 20 years losses of NO_3_^−^–N and NH_4_^+^–N observed were 1375.91 kg ha^−1^ and 9.24 kg ha^−1^, while in the simulation increase was from 1387.01 kg ha^−1^ and 9.28 kg ha^−1^ respectively. The average total change between observed and simulated NO_3_^−^–N and NH_4_^+^–N was about 11.15 kg ha^−1^and 0.04 kg ha^−1^ respectively. The losses of NO_3_^−^–N from soil were not the same for observed and simulated results by APSIM. It has directly related to the application of nitrogen rates and rainfall events. In general, the APSIM nitrogen model significantly predicted the changes in soil NO_3_^−^–N and NH_4_^+^–N over different application rates. The difference in precipitation significantly changes over-fertilization years, while essential soil water content also wildly fluctuates with rainfall events (R^2^ = 0.171). The denitrification in (Fig. 4) showed that the changes were significant with a year of fertilization in both observed and predicted. To study the impact of individual year, we divide the years into five scenarios and predicted the NO_3_^−^–N and NH_4_^+^–N losses with depth, denitrification, and nitrification with precipitation for observed and simulated values in the APSIM model. The average 20 years of losses of denitrification and urea losses in observed and predicted were not changed significantly. The essential soil water content remained the same and did not change over 20 years, as shown in Table 3.

### Nitrogen model on observed value year 1990

**Figure 5:**
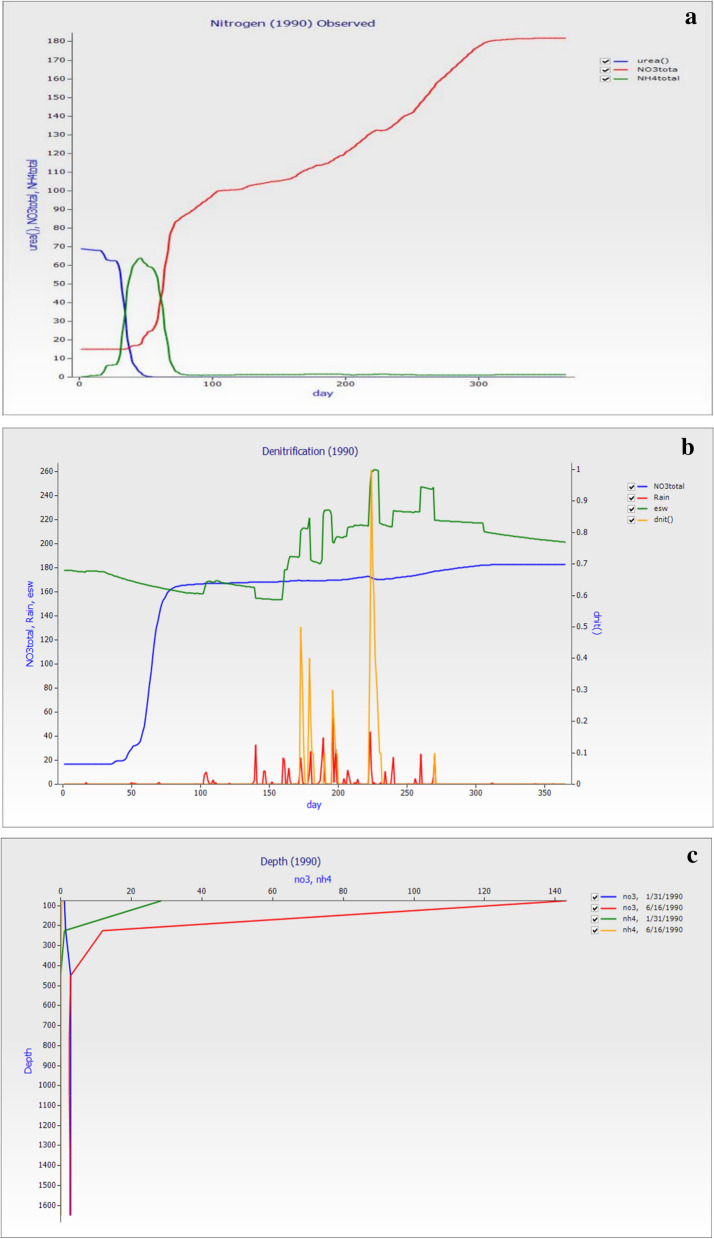
The graph was created for the year 1990 were (**a**) Date versus urea, total ammonium, and total nitrate, (**b**) Date versus Rain, DNIT (on Right Hand Axis), esw (extractable soil water) (mm), and total NO_3_^−^–N (kg ha^−1^) and total NH_4_^+^–N (kg ha^−1^) (**c**) distribution of nitrate through the soil profile throughout the depth.

The nitrogen model under climatic change (weather condition) on total nitrogen, denitrification, and depth nitrogen losses and simulation from 1990 to 2010. For every five years for observing data and simulation result for 1990 and 2010. The result in Fig. 5a showed that in the start of the days after urea application, the losses of the total NO_3_^−^–N and NH_4_^+^–N were minimized, while after 50 days in NH_4_^+^–N and NO_3_^−^–N on day 100, the losses were stated and increased up to 350. This is the change of losses of nitrogen in the soil after urea application. The graph in Fig. 5b showed the date versus rain, denitrification on the right-hand axis, essential soil water, and total NO_3_^−^–N. The graph showed that denitrification increased with the increase in rainfall. The average value of 365 days of esw was 192.9 mm, total NO_3_^−^–N was 114.01 mm, total NH_4_^+^–N was 6.32 mm, and dnit was 0.02 mm. The total NO_3_^−^–N also showed the same trend with rainfall and losses of it occurs with days’ increase, the losses of total NO_3_^−^–N (kg ha^−1^) in 7 layers was 48.47 followed by 24.92, 23.57, 49.60, 2.52, 2.39, and 2.45. The same decreasing trend was observed in total NH_4_^+^–N (kg ha^−1^) 5.29, 0.58, 0.28, and 0.140. The losses of both were minimum up to 50 days of fertilizer application while increase with days. The main cause of direct N_2_O emissions from agricultural fields was soil microbial activity, mainly nitrification (in well-aerated soils) and denitrification (in saturated soils) processes. Because these loss mechanisms are biological, soil N_2_O losses were caused by temperature and soil water conditions. Natural N_2_O emissions will occur as a result of whether fertilizer is used because soil organic matter decomposition often contributes to the same microbial soil processes that produce N_2_O. Nevertheless, the application of fertilizer would increase the amount of direct N_2_O emissions and indirect nitrogen losses significantly due to greater availability of N. The graph in Fig. 5c showed the distribution of nitrate through the soil profile on 31 January and 16 June after fertilization. The distribution of nitrate in the soil profile on days 1, 15 and 31 days after the addition of urea fertilizer and at 5 months. The result revealed that with depth on two different days of the year, simulation of leaching and losses of NO_3_^−^–N and NH_4_^+^–N occur. The losses increased with depth, the denitrification on 16th June is higher than the 31th January.

### Nitrogen result for the year 1995

**Figure 6:**
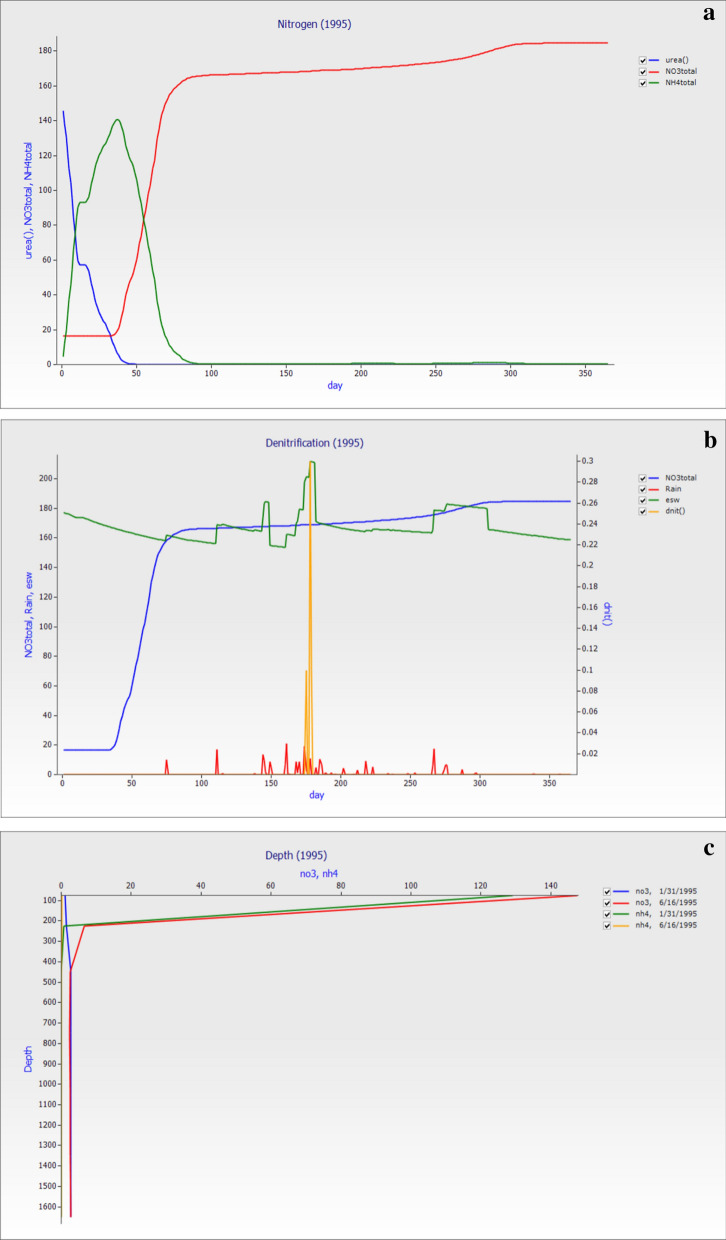
The graph of the year 1995 (**a**) between rain versus urea, NO_3_^−^–N and NH_4_^+^–N showed that the long-term fertilization effect on dynamic of nitrogen in the soil, (**b**) day versus total NO_3_^−^–N, Rain, Essential soil water (esw), total NH_4_^+^–N and rainfall in 1995 while (**c**). The losses (NO_3_^−^–N and NH_4_^+^–N) with different depths showed that NO_3_^−^–N losses depend on the rainfall events at different dates of the year.

Nitrogen directly or indirectly affected the metabolism and growth of plants in many aspects and was the main element affecting crop yield. In crop production, nitrogen and phosphorus fertilizers can improve and regulate soil nitrogen and phosphorus supply capacity and promote crop growth, which has become essential elements and means to increase crop yield. The graph in Fig. 6a was between rain versus urea, NO_3_^−^–N, and NH_4_^+^–N showed the long-term fertilization effect on the dynamic of nitrogen in the soil. The result was similar to the 1990 year simulation such as the urea vanish up to 50 days and soil NO_3_^−^–N was stable up to first fourty five days and start losses as it reached 50 days and the losses were high after this. The esw (mm) was 167.18, total NO_3_^−^–N (kg ha^−1^) was 110.57 and NH_4_^+^–N (kg ha^−1^) was 8.99, and dnit loses 0.4. The soil NH_4_^+^–N losses also occur after 85 days as depletion and leaching. This showed that the losses of nitrogen occur in the soil.

The soil nitrogen directly influences the crop growth and metabolism in the crop and one of the main elements in crop yield. The graph in Fig. 6b between day versus total NO_3_^−^–N, rain, essential soil water (esw) and total NH_4_^+^–N showed that rainfall in 1995 is relatively low and frequent rainfall occurs in the middle of the year. The total NO_3_^−^–N (kg ha^−1^) in the seven layers was 59.68 followed by 59.68, 23.31, 13.58, 6.63, 2.39, 2.45, and 2.52, the same decreasing trend was observed in total NH_4_^+^–N (kg ha^−1^) 8.11, 0.43, 0.30 and 0.14 respectively and zero up to 7th layer. The rainfall is the main factor in losses of nitrogen. When the soil is saturated with water, a process called denitrification and cause nitrogen was loss from the soil zone. Denitrification was the transformation of nitrate into one of the gaseous types of nitrogen that will be lost to the environment. This process usually occurs when there were conditions for the absence of oxygen (anaerobic), such as in saturated soils. A method called denitrification will cause a loss of N when the soil was saturated with water. Figure 6b showed denitrification losses between 150 and 200 days due to high rainfall between this time. When the long-term improper use of nitrogen fertilizer increases considerably, the accumulation of nitrate-nitrogen in deep soil, even more than the single application of nitrogen fertilizer.

The chance of leaching has increased considerably. To investigate the impact of nitrogen fertilizer on the . The nitrogen was lost through denitrification in large amounts of nitrate available in the soil when it’s in saturated conditions. Figure 6c showed that the losses of the total (NO_3_^−^–N and NH_4_^+^–N) with different depth showed that the losses of NO_3_^−^–N losses depend on the rainfall events; at 16-6-1995, the losses were higher as compared with the January 31. The NH_4_^+^–N also followed the same pattern because it was also affected by rainfall and temperature. Compared with the soil layers, the change of the total nitrogen content with the soil layer depth gradually increased. This indicates that with the increase of nitrogen application rate, the accumulation of soil nitrogen below the root layer (generally, the corn root layer is 120 cm) increases, which increases the leaching loss of nitrogen.

### Nitrogen dynamics for the year 2000

**Figure 7:**
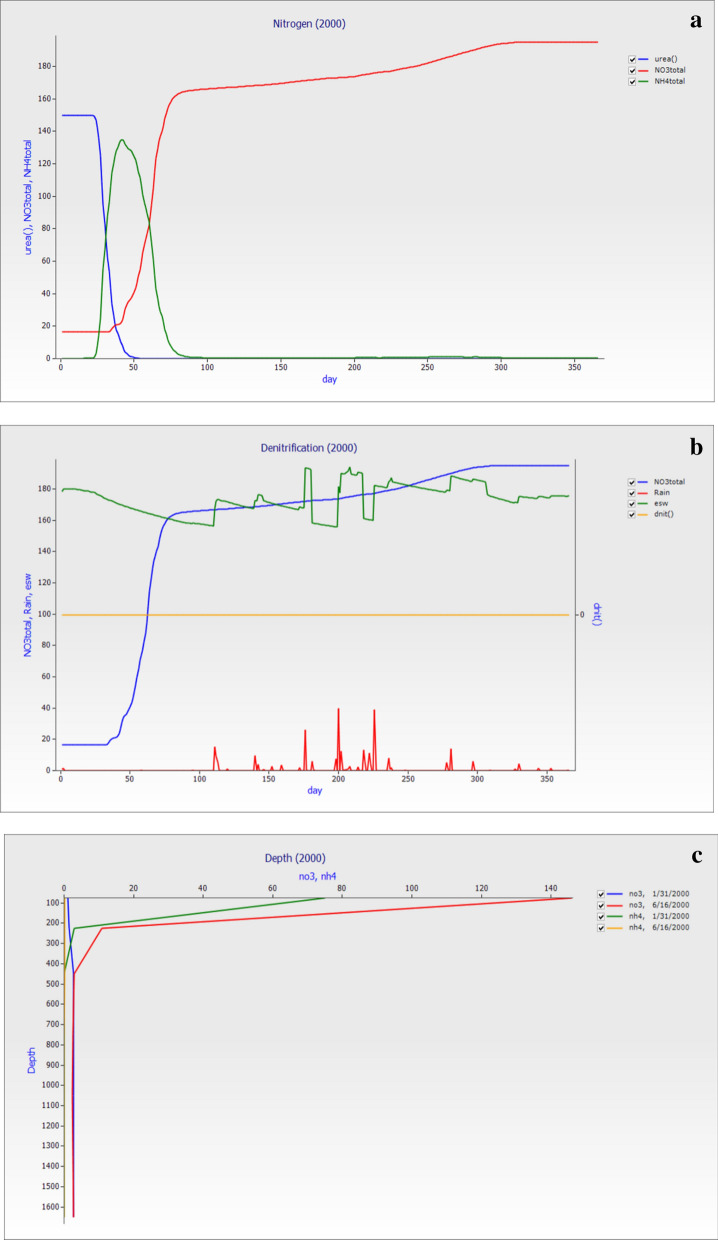
The graph of the year 2000 (**a**) the graph between Rain versus urea, Total NO_3_^−^–N and NH_4_^+^–N and days of the year (**b**) day versus Total NO_3_^−^–N, Rain, Essential soil water (esw) and Total NH_4_^+^–N while (**c**) The losses (NO_3_^−^–N and NH_4_^+^–N) with different depth indicated that the losses of NO_3_^−^–N losses depend on the rainfall occasions at other dates of the year 2000.

The results in Fig. 7a showed that in the year 2000, the losses of nutrients were the same and followed the 1995 year with minimum variation. The rainfall event is also high from 150 to 220 days of the year, so the essential soil water also fluctuates, which results in the changes in total NO_3_^−^–N in the soil. The average mean of esw was 311.53 mm, total NO_3_^−^–N was 1359.93 kg ha^−1^_,_ NH_4_^+^–N was 16.77 kg ha^−1^ and dnit was 0.03 kg ha^−1^_,_ respectively. The total NO_3_^−^–N in kg ha^−1^ in first seven layers were 320.82, 192.44, 356.21, 265.76, 139.55, 61.65, and 23.5, while NH_4_^+^–N were 16.04, 0.27, 0.28, and 0.17. The losses started when rainfall occurs, and the denitrification showed a significant change in Fig. 7b. The Total NH_4_^+^–N denitrification in January was higher as compared to June. The denitrification losses in this year were less than in previous years because of low rainfall. While in Fig. 9c showed that the changes of the depth versus different dates wise. The NO_3_^−^–N and NH_4_^+^–N losses showed that the change was slightly different from the 1995 pattern. In Fig. 7c, the total NO_3_^−^–N losses in June-16 started higher losses with depth than January-31.

### Nitrogen changes in 2005

**Figure 8:**
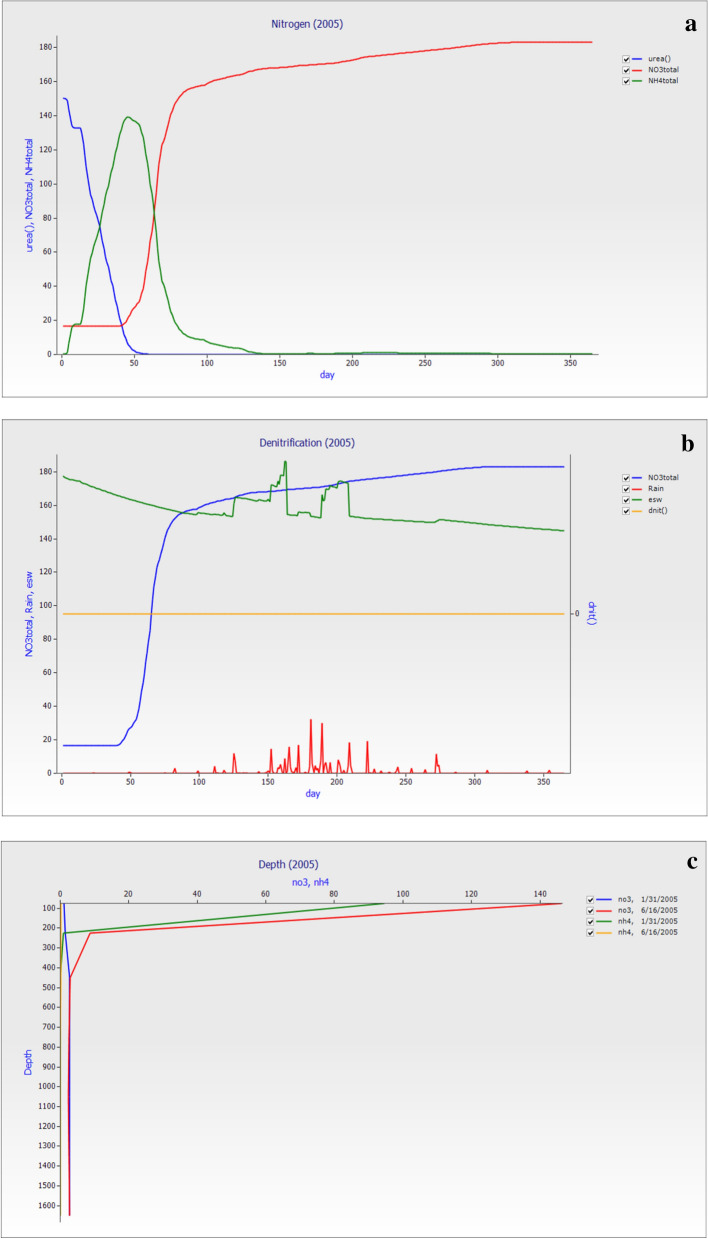
The graph was made on simulation datausing nitrogen model for the year 2005 (**a**) the nitrogen changes graph with different days of the year versus urea, total (NO_3_^−^–N and NH_4_^+^–N), (**b**) the graph of day versus total NO_3_^−^–N, Rain essential soil water (esw) and denitrification (dnit) while, (**c**) the graph of nitrate distribution through the soil depth at different dates in the year.

The nitrogen model simulation through APSIM was shown in Fig. 8a, the urea, total NO_3_^−^–N and NH_4_^+^–N versus days in the year 2005 simulation of nitrogen changes as urea fertilizer was applied. The losses of the total NO_3_^−^–N and NH_4_^+^–N were followed by the year 2000. The losses were similar to the previous year. The esw was 157.20 mm, total NO_3_^−^–N was 115.70 kg ha^−1^, total NH_4_^+^–N was 8.48, and zero denitrification. The average total NO_3_^−^–N (kg ha^−1^) losses were 63.15, 20.45, 16.28, 8.38, 2.42, 2.47, and 2.56 while total NH_4_^+^–N losses in (kg ha^−1^) was 7.74, 0.33, 0.25, and 0.13 respectively. Figure 8b showed that the day versus total NO_3_^−^–N, rainfall, essential soil water (esw), and denitrification (dnit) showed that the rainfall was high in the mid-year range from 150 to 215 days. The total soil NO_3_^−^–N losses were highly dependent on the rainfall event. The essential soil water also changes with the rainfall and constant when rainfall did not occur in the start and end of the year. The total soil NO_3_^−^–N losses were high, and Fig. 8c showed that the Nitrate distribution through the soil profile at 31 days after fertilization and again at 5 months. To help visualize this, we build a depth plot. Layered variables were kept in ranges at all times. For this reason, we included NO_3_^−^–N and NH_4_^+^–N as layered variables, and the nitrogen leach downward up to the soil depth. Figure 8c showed the distribution of nitrate in the soil profile after 21 days of fertilizer addition and at 5 months.

### Nitrogen dynamics in 2010

**Figure 9:**
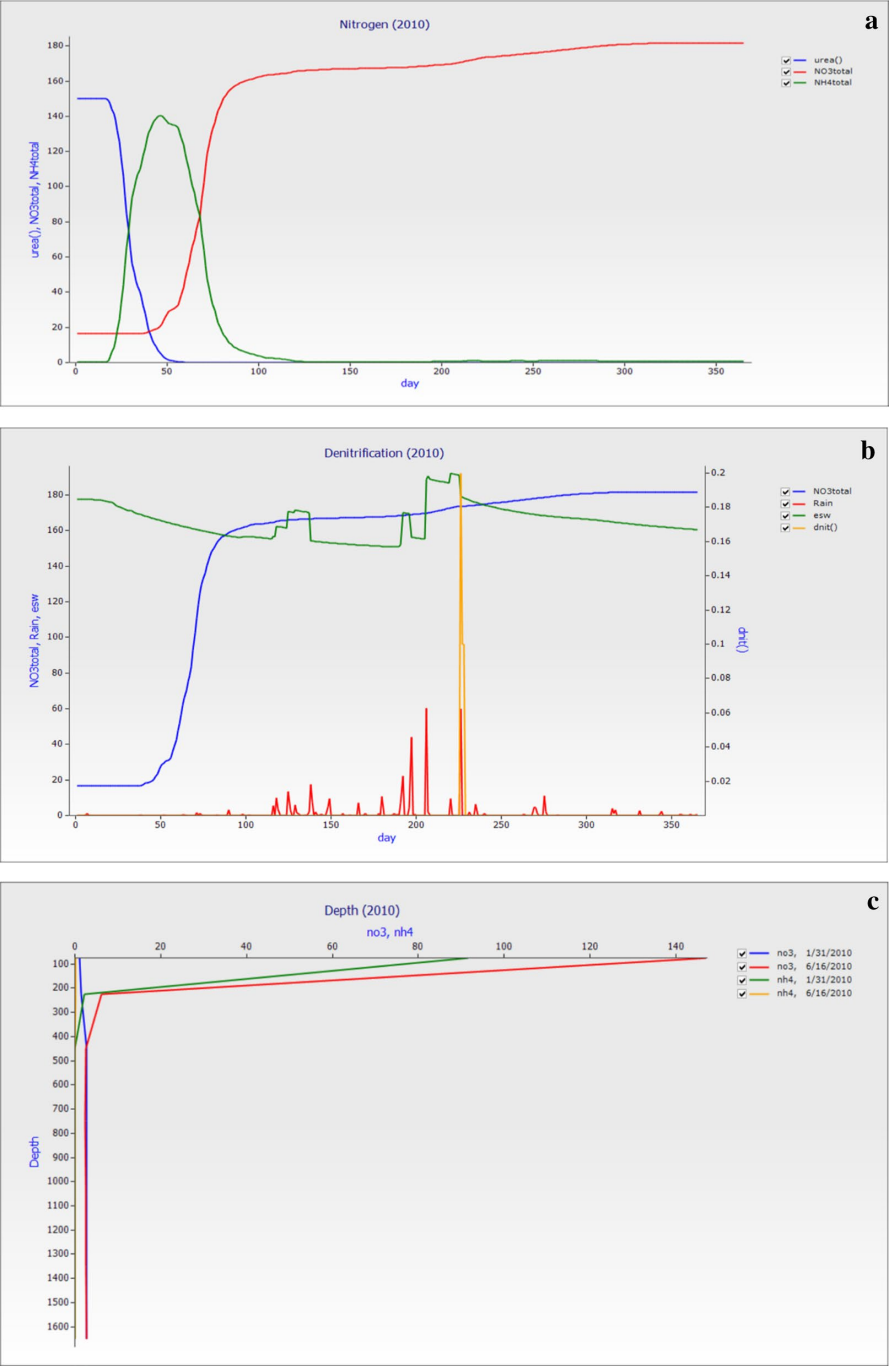
The graph was made on observed data for the year 2010 (**a**). The soil nitrogen Total (NO_3_^−^–N, and NH_4_^+^–N) and urea versus day. (**b**) The days of the years versus denitrification (dnit), rainfall, esw, and total NO_3_^−^–N while (**c**) depth of the profile versus NO_3_^−^–N and NH_4_^+^–N with different year dates.

The losses of nitrogen through leaching from agricultural soil leads to low yield and environmental issues. The soil total nitrogen (NO_3_^−^–N and NH_4_^+^–N) and urea versus day were shown in Fig. 9a; it states that, after urea fertilization, the soil total (NO_3_^−^–N and NH_4_^+^–N) losses were minimum up to the first 50 days of the year of fertilization while increase occurs as days passes and showed constant losses up to the end of the year. The average annual mean of esw was 165.37, total NO_3_^−^–N was 108.73 kg ha^−1^, NH_4_^+^–N was 8.03 kg ha^−1^, and dnit was 0.7 kg ha^−1^ respectively. The total NO_3_^−^–N (kg ha^−1^) were 55.5, 22.24, 16.23, 7.32, 2.42, 2.47, and 2.52 and total NH_4_^+^–N (kg ha^−1^) were 7.06, 0.54, 0.27, and 0.13. The losses in Fig. 9b of total NO_3_^−^–N showed that the losses were higher than previous years and the rain fall in this year was higher, which affected the soil saturation level and led to nitrogen losses. The denitrification (dnit) losses were higher among 200–250 days, and rainfall was also high. Soil essential water also fluctuated with the rainfall throughout the year. Figure 9c showed that the nitrogen losses with depth occur in the soil, and the graph showed that the NO_3_^−^–N losses were higher in the 5th month of the year compared to the start of the year. The NH_4_^+^–N losses also suggest that the losses were higher on the 5^th^ of the month than the start of the month. This showed that characteristics of nitrate leaching in this region occurred, and it needs to address during the summer maize season.

### Simulated data results for the years 1990 and 2010

#### Nitrogen simulation on simulated data

**Figure 10:**
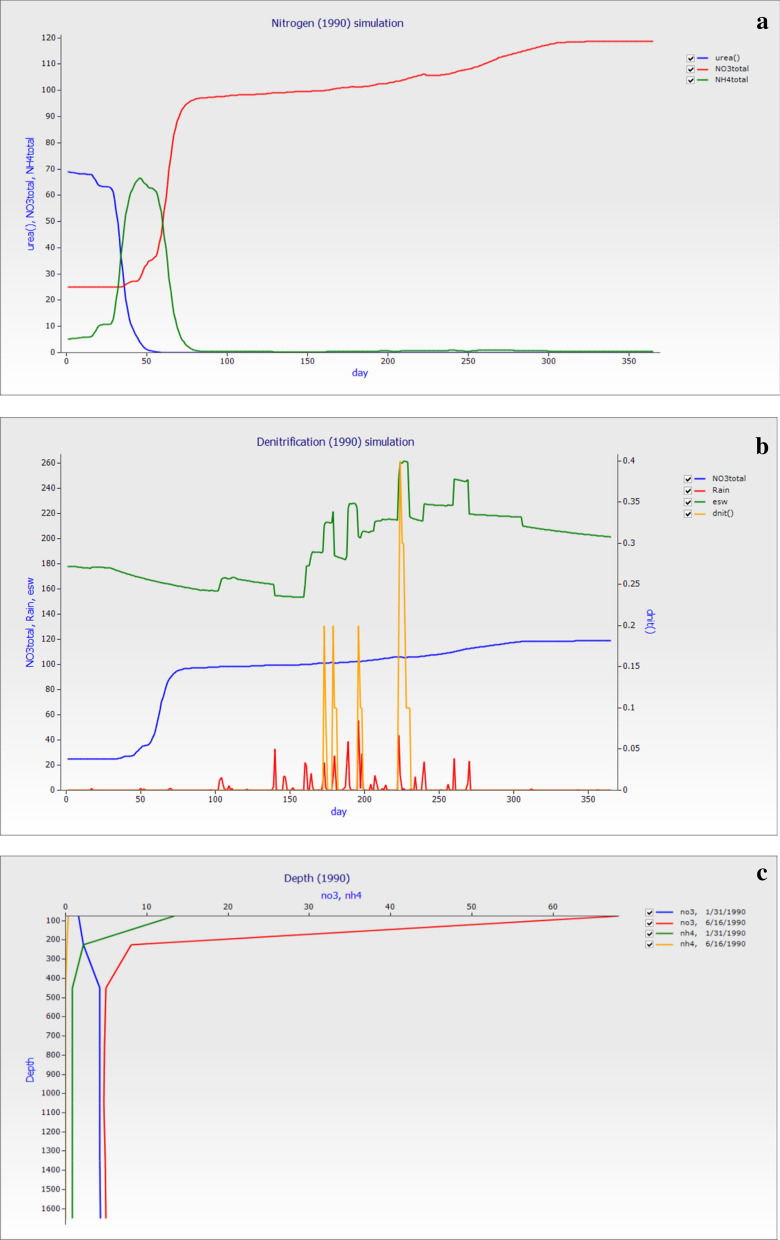
The graph was created on weather data of 1990, and the simulation of NO_3_^−^–N and NH_4_^+^–N value was change to check the simulation of nitrogen losses with the same weather data. (**a**) nitrogen simulation graph such as days versus total (NO_3_^−^–N and NH_4_^+^–N) and urea, (**b**) denitrification graph in which days of the years versus denitrification (dnit), rain, esw, and total NO_3_^−^–N while, (**c**) the depth of the profile versus NO_3_ and NH_4_^+^–N with different days of the year.

To check the dynamics of nitrogen level, if the nitrogen level increase in the soil from the observed value, what will be the fate of nitrogen. The nitrogen simulation in Fig. 10a was made on the same environmental condition; soil properties and all other conditions were kept constant. The change in total NO_3_^−^–N and NH_4_^+^–N were change from 25 kg ha^−1^ to 5 kg ha^−1^. The average simulated observation for esw was 192.92 mm, total NO_3_^−^–N was 128.07 kg ha^−1^, total NH_4_^+^–N was 7.17 kg ha^−1^, and dnit was 7.7 kg ha^−1^. The average annual losses of total NO_3_^−^–N (kg ha^−1^) were 49.27, 25.87, 26.09, 12.16, 4.97, 4.81, and 4.87 while NH_4_^+^–N kg ha^−1^ were 5.35, 0.65, 0.42, 0.28, 0.13, 0.14, and 0.14 respectively. In Fig. 10a Total NO_3_^−^–N showed that losses were high after 50 days, and higher losses were observed up to the end of the year. If large amounts of nitrate were available in saturated soil conditions, nitrogen was lost through denitrification. Soil essential water requirement and denitrification were related to each other; higher denitrification occurs between 150 and 240 days of the year. The higher denitrification was in 200–250 days, as shown in Fig. 10b. An increase in precipitation also enhanced the denitrification rate in the soil. The NH_4_^+^–N losses were up to 400 m depth while NO_3_^−^–N losses started from 100 m depth after 31^th^ month of fertilization, as shown in Fig. 10c. The NH_4_^+^–N start losses up to 0.32% till the soil depth while NO_3_^−^–N losses started from 65 till 600 m up to the soil depth.

**Figure 11:**
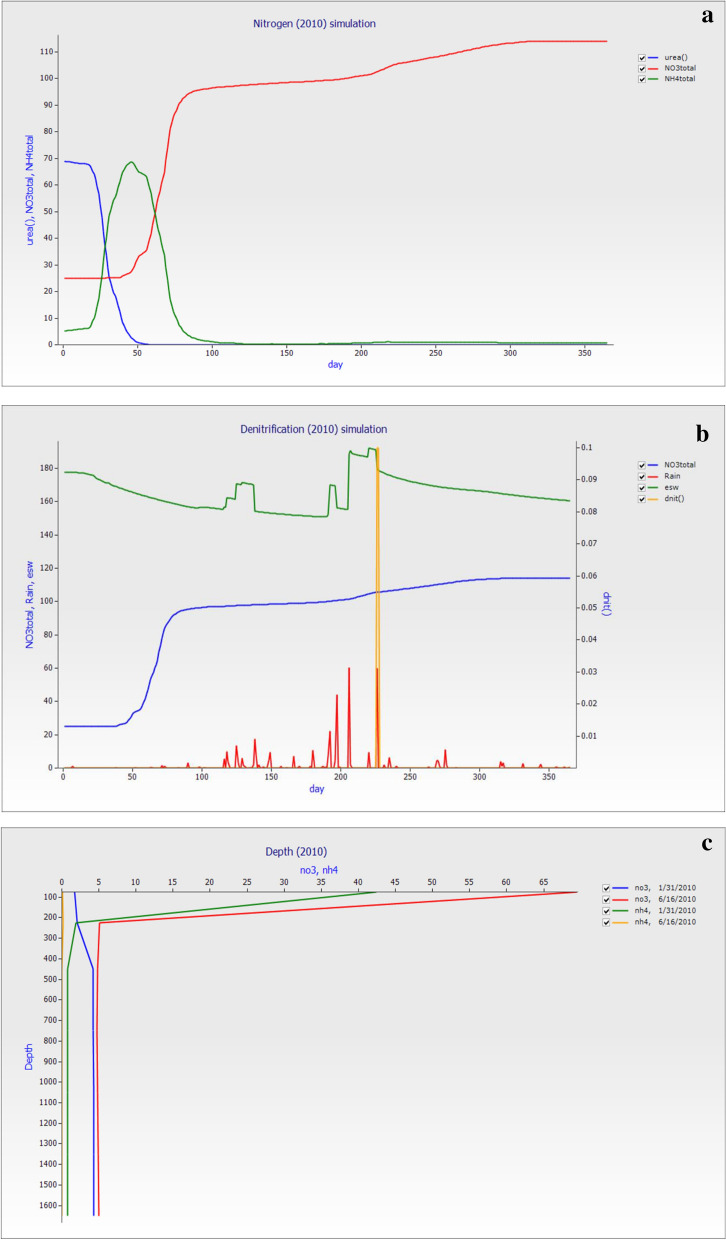
The graph was generated on simulated data for the year 2010 (**a**). The soil nitrogen Total (NO_3_^−^–N and NH_4_^+^–N) and urea versus day. (**b**) The denitrification (dnit), rainfall, esw, and total NO_3_^−^–N versus days of the year’s while (**c**) The depth of the soil profile NO_3_^−^–N and NH_4_^+^–N with two different dates of the year.

The nitrogen simulation in Fig. 11a showed that after urea fertilization, it depletes in the soil after 50 days. This result in the uptake of nitrogen by the plant was high at the start of the year. The total NO_3_^−^–N showed that losses started from 80 days of the year while NH_4_^+^–N losses started after 100 days. The average mean of annual esw was 165.37 mm, total NO_3_^−^–N was 122.48 kg ha^−1^, total NH_4_^+^–N was 8.96 kg ha^−1^, and dnit was 0.7 kg ha^−1^. The annual average mean of seven layers of NO_3_^−^–N (kg ha^−1^) losses were 56.51, 23.27, 18.6, 9.65, 4.74, 4.80, and 4.85 while total NH_4_^+^–N (kg ha^−1^) were 7.12, 0.61, 0.42, 0.29, 0.15, 0.15, and 0.15. The rainfall in Fig. 11b was constant, and this year receive maximum rainfall and reached up to 60 mm at peak level on 210 days of the year. The essential soil water (esw) showed a different level throughout the year. The denitrification rate only experienced on 220 days of the year. At the same time, total NO_3_^−^–N was available in the start-up to 80 days while start depletion. The soil depth Fig. 11c represents that in the start 31th January, the NO_3_^−^–N show losses up to 1000 mm while NH_4_^+^–N loss started from 50 to 400 m. After the 5th month of fertilization application, the depletion of NH_4_^+^–N showed depletion from 0.1 to 0.2 and sudden change and losses started. The NO_3_^−^–N started from 65 to downward up to 200 cm and below the root zone.

## Discussion

The globe's average temperature increased over the last decades and continues to increase and rise in prediction, with the great chance to experience hot days—this increase in temperature solar radiation, and precipitation will have a high impact on agriculture^47^. The world's annual fertilizer nitrogen consumption has reached up to 70 million tons. China has an annual consumption of over 15 million tons and is the world's largest consumer of fertilizer nitrogen.

The result of simulation indicates that for enhancing crop production and APSIM mineralization, the chemical composition of biochemical needs to address and accounted for, moreover by enhancing the soil N pool from crop residues which come from C and N partition (CARB, CELL etc.), the crop biochemical composition of residual crop or by adding a conceptual pool or imitated SMM. Various studies also support the result by separating the chemical digestion of organic material into fractions^48,49^. However, nitrogen fertilizer efficiency was low, and there were large losses to the environment. It is estimated that agricultural nitrogen losses can be as high as 40–60% of nitrogen in our country^50^. Overuse of chemical N fertilizers, high net mineralization and nitrification, and predominance of rainfall during the summer season with a light soil texture were the main control factors responsible for the heavy nitrate leaching^51^. In this study, we assessed the dynamic of nitrogen fertilization and climate change with respect to solar radiation, temperature, precipitation, and its impact on nitrification, denitrification, and nutrient losses with depth. We used the nitrogen model in APSIM and used irrigation level constant to check the soil runoff, which plays a key role in nitrate leaching, depending primarily on precipitation and irrigation levels. For example, when drainage declined from 570 to 79 mm, the irrigation rate decreased from 500 mm to no irrigation^52^. Water was an important feature in nitrogen losses, and nitrate was transported by the flow of soil water and can lead to loss of leaching if there were abundant water movement out of the root zone. Leaching of nitrates also occurs during the drainage season when precipitation and irrigation surpass evaporation^53^. The result was similar to the finding of^54^ losses of nitrogen included leaching of nitrate (NO_3_^−^–N) and gaseous emissions via ammonia (NH_3_) volatilization and denitrification (emissions of nitrous oxide (N_2_O) and dinitrogen (N_2_). In this study, the nitrogen model uses rainfall parameters to check the denitrification, the higher the rainfall higher will be the denitrification. Our result was similar to the findings of^55^, which stated that pasture growth in the deep soils was not affected by irrigation frequency, while denitrification increased with higher frequency irrigation, particularly in the poorly drained soil, resulting in increased N_2_O emissions. All soils and climates showed significantly higher denitrification and N_2_O emissions under high-frequency/low-intensity irrigation (Irr1) compared to low-frequency/high-intensity (Irr6), because soils exceeded a critical moisture content that favors denitrification. The rainfall cause runoff, and it result in losses of nitrogen; our result was also similar to the finding of^56,57^ where he stated that nitrogen losses by leaching and surface runoff were the highest among all climatic scenarios for each treatment under the RCP 8.5 scenarios. The explanation may be due to the expected more regular heavy precipitation events and high soil nitrate concentration due to the introduction and mineralization of mineral Nitrogen. In our study, the higher average annual denitrification rates under some years under APSIM scenarios, this result was similar to the finding of^56,58^, he stated that the climate change would increase N_2_O emissions globally. Based on the simulating findings in Fig. 11, inter annual variability steadily enhanced application of nitrogen rates greater than 25 kg N ha^−1^ similar result was also reported by^59–61^. The Nitrogen budget or balance was often calculated by comparing different N inputs and outputs in plant–crop systems, taking into account shifts in soil mineral Nitrogen^57^. In most cases, in spite of the great uncertainty associated with its calculation, denitrification was also seen as an important process of nitrogen loss. A long-standing issue in soil N research was the direct quantification of denitrification nitrogen loss from nitrogen fertilized soils^57^. This result was similar to the findings of our nitrogen model simulation of nitrogen losses; for example, willigen P compared 14 nitrogen cycle models and found that these 14 models could not be simulated in late spring and early summer. Loss of soil inorganic nitrogen after fertilization^62^. However, the study provided the adverse effects of climate change and give a clear picture of nitrogen dynamics under long-term weather conditions. In most cases, despite the great uncertainty associated with its measurement, denitrification was also seen as an important process of nitrogen loss. Clear denitrification quantification, the loss of nitrogen from N-fertilized soils were a long-standing issue in soil nitrogen research^57^. Because the soils were above a critical moisture content that favors denitrification^55^. Yet expected N losses have increased by leaching, denitrification, and N_2_O emissions. Pasture growth in the deep soils was not affected by irrigation frequency, while denitrification increased with higher frequency irrigation, especially in the poorly drained soil, resulting in increased N_2_O emissions. Based on these modeling results, it was possible to reduced nitrogen losses with little effect on^54^. The change in the soil microsites under the incubation conditions may also illustrate the response of N_2_O of emissions from denitrification response to the availability of temperature and soil NO_3_^−^–N. As observed, the increase in the temperature of soil respiration levels is likely to result in O_2_ depletion, affecting NO_3_^−^–N as the terminal electron acceptor during denitrification. The nitrous oxide emission from denitrification and partitioning of gaseous losses as affected by nitrate^63^.

The study indicated that the use of synthetic nitrogen (N) fertilizer has played a critical role in boosting food production to an increasingly growing population of the world. Furthermore, high inputs of mineral N fertilizer overcrop demands can lead to decreased N use efficiency and affect several negative impacts on the environment, such as surface water eutrophication, groundwater nitrate pollution releasing greenhouse gases, and soil acidification. These environmental issues were getting worse because the use of mineral nitrogen fertilizer would grow predictably in the future^19^. Complex interactions between soil properties, weather patterns, crop growth, and nitrogen loss networks make it more challengingto sync up fertilizer management with crop nitrogen demand, leading to under- or above-N utilization^64^. As such, the overall objective of this study was to provide a clearer understanding of increasing dynamics in the supply and demand balance of water, its effects on food production^17^. Appropriate strategies for fertilization must be adopted for further optimization to fit in with future climate change. Future more, climate change could significantly impact the soil nitrogen, especially the combined effects of elevated temperature, increased losses, and increased precipitation event increase denitrification. The APSIM nitrogen simulation model was used to simulate the influence of climate change on soil nitrogen dynamics based on future climate change across maize cropping regions in China.

## Conclusion

Quantitative information on nitrogen and its impact on soil under long-term fertilization, the best strategies, is essential for the assessment of nitrogen loss and availability. The results of the model showed that the nitrogen model between observed and simulated values for nitrogen losses, denitrification, and nitrogen losses through different depths. The nitrogen simulation through APSIM showed that after urea fertilization, it depletes in the soil after 50 days. This result in the uptake of nitrogen by the plant was high at the start of the year. The total NO_3_^−^–N showed that losses started from 80 days of the year while NH_4_^+^–N losses started after 100 days. The model predicted that with an increase in rainfall events in the year, losses of nitrogen increase. This study illustrates the potential for using crop management and nitrogen simulation models as an information technology tool for maintaining the suitable management strategies for maize production in Jilin province, China. This can provide alternative management strategies to overcome the nitrogen losses in maize crops. The weather record and long-term soil data offer the best management scenario for the analysis and prediction of nitrogen. The future recommendation about the study area was to 20-year, long-term simulation with APSIM validated model exhibited that N application (15 kg N ha^−1^) improves both the long-term average nitrogen losses. The nitrogen content 25 (kg ha^−1^) showed increased N losses with the same climatic condition. This implies a lower average yield under an increased amount of nitrogen application; hence 15 kg N ha^−1^ appear more appropriate for farmers, therefore and a higher yield with minimum losses of N will be observed, thus vulnerability will be reduced.

## Supplementary Information

Supplementary Information.
