# Supplementary material for: 20 Years nitrogen dynamics study by using APSIM nitrogen model simulation for sustainable management in Jilin China
Source: Sci Rep. 2021 Sep 1;11:17505. doi: 10.1038/s41598-021-96386-5 (PMC8410903; doi:10.1038/s41598-021-96386-5)
Supplement: Supplementary file 1 — Supplementary Information. [file 41598_2021_96386_MOESM1_ESM.docx]

20 Years Nitrogen dynamics study by using APSIM Nitrogen Model simulation for sustainable management in Jilin China

Nazia Tahir ^1,2^, Jumei Li ^1^, Yibing Ma^1,3^, Aman Ullah^1^, Ping zhu^4^, Chang Peng^4^, Babar Hussain^1^ and Subhan Danish^5^

**Supplementary Table**

**S.1.** Soil depth water content, lower limit for plant available soil water; DUL: drained upper limit; OC: soil organic carbon in soil.

| **Depth**  **(mm)** | **SAT** | **DULL**  **(%)** | **LL 1.5 (%)** | **Air Dry**  **(mm/mm)** | **OC**  **(kg kg^−1^)** | **Fbiom** | **Finert** |
| --- | --- | --- | --- | --- | --- | --- | --- |
| **0-10** | 59 | 47 | 0.19 | 0.04 | 1.2 | 0.02 | 0.6 |
| **10-20** | 49 | 39 | 0.2 | 0.14 | 1.0 | 0.01 | 0.65 |
| **20-40** | 49 | 39 | 0.2 | 0.15 | 0.86 | 0.01 | 0.7 |
| **40-60** | 49 | 39 | 0.2 | 0.16 | 0.83 | 0.01 | 0.75 |
| **60-80** | 49 | 39 | 0.2 | 0.18 | 0.58 | 0.01 | 0.8 |
| **80-100** | 49 | 39 | 0.2 | 0.18 | 0.54 | 0.01 | 0.85 |
| **100-120** | 49 | 39 | 0.2 | 0.18 | 0.47 | 0.01 | 0.93 |
| **120-140** | 49 | 39 | 0.2 | 0.18 | 0.5 | 0.01 | 0.93 |
| **140-160** | 49 | 39 | 0.2 | 0.18 | 0.5 | 0.01 | 0.93 |
| **160-180** | 49 | 39 | 0.2 | 0.18 | 0.5 | 0.01 | 0.93 |

**S.2.** Initial soil property of Jilin

| **Soil Horizon** | **Soil Layer** | **Organic Matter** | **Total Nitrogen** | **Total Phosphorous** | **Total Potassium** | **Available Nitrogen** | **Available Phosphorous** | **Available Potassium** | **pH** |
| --- | --- | --- | --- | --- | --- | --- | --- | --- | --- |
|  | **(cm)** | **(g/kg)** | **(g/kg)** | **(g/kg)** | **(g/kg)** | **(mg/kg)** | **(mg/kg)** | **(mg/kg)** | **-** |
| Aa | 0-20 | 20 | 1.34 | 0.546 | 16.355 | 153 | 10 | 119 | 7.6 |

**S.3.** Initial Soil Organic Matter Status calculated by APSIM nitrogen model

| **Layer** | **Hum-C** | **Hum-N** | **Biom-C** | **Biom-N** | **FOM-C** | **FOM-N** |
| --- | --- | --- | --- | --- | --- | --- |
|  | **kg/ha** | | | | | |
| **1** | 15861.2 | 1321.8 | 188.8 | 23.6 | 138.9 | 3.5 |
| **2** | 10045.6 | 837.1 | 79.4 | 9.9 | 108.1 | 2.7 |
| **3** | 10105.0 | 842.1 | 20.0 | 2.5 | 65.6 | 1.6 |
| **4** | 8092.0 | 674.3 | 8.0 | 1.0 | 39.8 | 1.0 |
| **5** | 4050.0 | 337.5 | 0.0 | 0.0 | 24.1 | 0.6 |
| **6** | 4050.0 | 337.5 | 0.0 | 0.0 | 14.6 | 0.4 |
| **7** | 4050.0 | 337.5 | 0.0 | 0.0 | 8.9 | 0.2 |
| **Total** | 56253.7 | 56253.7 | 56253.7 | 56253.7 | 56253.7 | 56253.7 |

**S.4.** Soil properties

| **Depth** | **Rock (%)** | **Texture** | **Munsell Colour** | **EC**  **(1:5 ds/m)** | **pH**  **(1:5 water)** | **ESP**  **(%)** |
| --- | --- | --- | --- | --- | --- | --- |
| **0-15** | NIL | clay loam | black | 0.100 | 7.6 | 1.000 |
| **15-30** | NIL | clay loam | black | 0.200 | 7.6 | 2.000 |
| **30-60** | NIL | clay loam | black | 0.200 | 7.6 | 3.000 |
| **60-90** | NIL | clay loam | black | 0.300 | 7.6 | 5.000 |
| **90-120** | NIL | clay loam | black | 0.400 | 7.6 | 8.000 |

**S.5.** Climatic properties of Jilin calculated by APSIM

| **Jilin** | | |
| --- | --- | --- |
| **Climate factors** | Tave (°C per 20 years) | 5.82 |
|  | Tmax (°C per 20 years) | 12.27914 |
|  | Tmin (°C per 20 years) | -0.50893 |
| **Represent the region with** | Total P (mm per 20 years) | 0.98567 |
|  | SR (MJ m-2 day-1 per 20 years | 653.7012 |
|  | amp (°C) | 39.5 |

b
